# Acute Appendicitis in Pregnancy and the Developing World

**DOI:** 10.1155/2017/2636759

**Published:** 2017-07-20

**Authors:** Tika Ram Bhandari, Sudha Shahi, Sarita Acharya

**Affiliations:** ^1^Department of General Surgery, Universal College of Medical Sciences, Bhairahawa 32900, Nepal; ^2^Department of ENT, National Academy of Medical Sciences, Kathmandu 44600, Nepal; ^3^Department of Obstetrics and Gynecology, Universal College of Medical Sciences, Bhairahawa 32900, Nepal

## Abstract

**Background:**

Acute appendicitis is the commonest nonobstetric surgical emergency during pregnancy. The aim of the study was to compare perioperative outcomes of acute appendicitis in pregnant and nonpregnant patients.

**Methods:**

A retrospective review of medical records of 56 pregnant patients between 2011 and 2016 who were compared with 164 nonpregnant women of reproductive age who underwent open appendectomy between 2014 and 2016 for acute appendicitis. The patient's demographics and perioperative data were analyzed.

**Results:**

The median age of pregnant and nonpregnant patients observed was 26 years (range 19–37) and 26 years (range 18–43). There were no significant differences between the groups in negative appendectomy (21.4 and 21.3%, *P* = 0.52), perforated appendicitis (25 and 23.8%, *P* = 0.85), postoperative complications (28.6 and 26.8%, *P* = 0.80), and median length of hospital stay (5 and 4.5 days, *P* = 0.36). There were 3.6% preterm labour, no maternal mortality, and no fetal loss. In multivariate analysis, WBC >18000/mm^3^ and long patient time to surgery were independent risk factors for appendicular perforation and postoperative complication (*P* < 0.05).

**Conclusion:**

Our results of appendectomy in pregnant patients are comparable with nonpregnant patients. Hence the same perioperative treatment protocol can be followed in pregnant and nonpregnant patients even in resource-poor setting.

## 1. Introduction

Acute appendicitis is the commonest nonobstetric emergency requiring surgical intervention in a pregnant patient [1]. Symptoms and signs of pregnancy are variable among the patients [2, 3]. Abdominal ultrasonography may have a low accuracy and computed tomography is usually avoided in pregnancy as much as possible, mostly in the first trimester, due to hazards of exposure to ionizing radiation [1, 4]. Meanwhile, accurate diagnosis is essential during pregnancy to reduce negative appendectomy. Delay in diagnosis and treatment also results in increased risk of developing perforation which can lead to poor postoperative outcomes [5, 6]. There are only few studies [7, 8] that have been reported from developing countries about acute appendicitis in pregnant patients until now. The exact rate of acute appendicitis in pregnant women is not identified and its management is imprecise in a developing nation like ours. The aim of this study was to compare clinical presentation and perioperative outcomes of acute appendicitis in pregnant and nonpregnant patients in resource-poor setting.

## 2. Material and Methods

A retrospective review of medical records of 56 consecutive pregnant patients who underwent open appendectomy between 2011 and 2016 was done. The data was compared with that of 164 nonpregnant female patients of reproductive age, who had undergone open appendectomy between 2014 and 2016 at Universal College of Medical Sciences, Bhairahawa, Nepal. The study was approved by the Institutional Review Committee of Universal College of Medical Sciences, Bhairahawa, Nepal. The gestational period was categorized as the first (0–13 weeks), second (14–26 weeks), and third trimester (27 weeks and beyond). The reproductive age has been defined between 15 and 49 years according to World Health Organization (WHO). Time to surgery was defined as the period from onset of symptoms till surgery. Abnormal WBC was considered less than 4000/mm^3^ (leukopenia) or more than 11000/mm^3^ (leukocytosis). Leucocytosis was again subdivided into less than 18000/mm^3^ and more than 18000/mm^3^ since mild to moderate leukocytosis are features common to both normal pregnancy and acute appendicitis. Temperature above 37.8°C was considered as fever. A diagnosis of acute appendicitis was made based on clinical presentation and laboratory and radiological findings.

For patients in earlier weeks of pregnancy, we followed them up till 30th postoperative day after appendectomy. Surgical outcomes were recorded during that period. After that the patients followed up on pregnancy outcome in obstetric department. In case of the patient who had labor pain shortly after appendectomy, she was shifted to obstetric unit at our hospital and delivery was conducted. Coordination with the obstetric team made us easy for follow-up of these patients during perioperative period. Records were maintained. The patient's preoperative, operative details, postoperative outcomes, and pregnancy related outcomes were analyzed.

All continuous variables were expressed as median or mean ± standard deviation which were compared with Mann-Whitney test or independent *t*-test, as appropriate. Chi-square test or Fisher's exact test was used for categorical values as appropriate. Perforated appendicitis and postoperative complications were the outcome variables in the study. Multivariate analysis (logistic regression) was considered for those variables that were significant for outcome variable on univariate analysis. All data were analyzed using SPSS version 22.0 for Windows. *P* value < 0.05 was considered as statistically significant.

## 3. Results

Fifty-six pregnant patients with median age 26 years (range 19–37) and 164 nonpregnant female patients of reproductive age with median age 26 years (range 18–43) who underwent open appendectomy for acute appendicitis during the study period were studied. Out of the 56 pregnant patients, 23 (41.1%) patients had surgery during the first trimester, 26 (46.4%) during the second trimester, and 7 (12.5%) during the third trimester. Most of the pregnant patients were multipara 36 (64.3%). Right lower quadrant pain of the abdomen was the most common clinical symptom irrespective of the gestational period in pregnant patients and nonpregnant patients. Patient's preoperative data are shown in Table 1. There were no significant differences in different preoperative variables (*P* > 0.05), when we compared these variables in pregnant and nonpregnant patients.

When we compared different intraoperative and postoperative variables in pregnant and nonpregnant patients, we did not find any significant differences in mean time to surgery, mean operative time, perforated appendicitis, the rate of complications, and negative appendectomy between the pregnant and nonpregnant groups (*P* > 0.05) which are shown in Table 2. The overall postoperative complications observed between the groups were 28.6 and 26.8%. Surgical site infection was the most common complication in both groups (14.3 and 13.4%). The pregnant patients had slightly longer median duration of hospital stay than the nonpregnant patients (5 and 4.5 days); however it was not statistically significant (*P* = 0.33). There was no mortality in either of the groups. Preterm labor occurred in 2 (3.6%) patients while postoperative fetal loss was not observed in our study.

There was no association of pregnancy status of patient with appendicular perforation and postoperative complications in acute appendicitis (*P* > 0.05). However, in multivariate analysis WBC > 18000/mm^3^ and patients time to surgery (onset of symptoms to surgery) were independent risk factors for appendicular perforations (OR: 47.9; 95% CI [13.68–168]; *P* = 0.001 and OR: 6.57; 95% CI [2.27–19.5]; *P* = 0.001) and for postoperative complications (OR: 11.1; 95% CI [4.09–30.4]; *P* = 0.001 and OR: 3.53; 95% CI [1.49–8.34]; *P* < 0.05), respectively.

## 4. Discussion

There is a paucity of data on acute appendicitis in pregnant patients from developing nations. Most of the previous studies stated that acute appendicitis presents atypically during pregnancy [9] and deserves an aggressive operative approach to prevent perforation and increased risk of poor outcomes [10]. However, recent upgrade in the precision of diagnostic radiological modalities and the effectiveness of antibiotic treatment has made the old approach questionable.

Several studies have reported the prevalence of acute appendicitis in pregnant population to be 0.06% to 0.28% [11, 12]. We found the incidence 1/800 (0.12%) of acute appendicitis during pregnancy which was comparable with other studies. Similar to what has been reported in the literature, most of our patients were in the second trimester [13]. However, Davoodabadi et al. [14] found that the incidence of acute appendicitis during pregnancy was high during the third trimester. The second trimester is considered as the most appropriate time for appendectomy and this period has the lowest risk for fetus from operation and anesthesia. Though the first trimester is the best time with respect to ease of operation, this time may be risky for the fetus. The third trimester is considered as the poorest time with respect to operative comfort and hazards of preterm birth. The median age of 26 years, in our study, is lower than that reported by some other studies [5, 15]. This could be due to an early marriage and early childbearing age in our part of the world as compared to the developed countries.

In our study, abdominal pain was the commonest symptom (100%) as reported by another study [16]. The pain typically migrates to the right iliac fossa and worsens when the patient coughs.

Usually pregnancy is related to leukocytosis in a healthy female, which is associated with increased number of neutrophils. In the second month of gestation, the neutrophil count starts to increase and plateaus in the second or third trimester. During that time the total leucocyte counts range from 10000 to 15,000/mm^3^ [14]. The current study reported 62.5% of abnormal leukocyte count which is in concordance with what was reported by Hiersch et al. [17]. There were no significant differences in WBC between the groups during admission. Ultrasonography may show distended nonperistaltic blind ended tubular structure appendix, but the appendix is seen in only 25–59% of patients with acute appendicitis. However, it is supportive in making diagnosis and exclusion of some other diseases [18]. In our series, acute appendicitis was detected in 44.6 and 44.5% of pregnant and nonpregnant patients by ultrasonography, respectively.

We reported negative appendectomy (21.4 and 21.3%, *P* = 0.52) similar to the negative appendectomy rate mentioned in the literature (23–50% and 15–35%) for a pregnant or nonpregnant cohort [19]. However, Zingone et al. specified 17.4% of negative appendectomy in their large population-based study [20].

There was no difference in the rate of perforated appendicitis between the pregnant and nonpregnant patients (25.0 and 23.8%, *P* = 0.85). The proportion of appendicular perforation in our study (25%) is higher than that previously reported [21]. It might be because our patients presented very late to the hospital thus delaying timely diagnosis and treatment [22]. Also, most of our patients were in the second or third trimester (58.9%). With growing gestational age diagnostic correctness decreases and is related to increased rates of appendiceal perforation and therefore postoperative complications.

We did not find any differences in postoperative complications between the groups (28.6 and 26.8%, *P* = 0.80). The complication rate 28.6% that we have found in pregnancy was comparable to those shown by other studies [23]. No maternal and fetal mortality was observed in spite of the high rate of perforation and high rate of complications. Improvement in maternal survival may be due to the use of antibiotics, improved anesthesia, and better care. However, a study reported 4-5% fetal loss rate after surgery [19]. Many studies have mentioned preterm delivery rate in pregnant patients with appendicitis up to 11.4% [24]. Two of our patients had preterm deliveries at 36th week of gestational age. Preterm labor occurred in the fourth and fifth hour of surgery, respectively, and both patients later underwent normal vaginal deliveries giving birth to healthy babies. It has been mentioned that the performance of any surgery during pregnancy increases the risk of premature labor by 10–15%. Both our patients had preterm delivery during the postoperative period after appendectomy while they were still in the hospital. So, there seems to be some association between preterm delivery and surgery in our cases also. In the present study we had only 3.6% (95% confidence interval; 0.4–12.3%) of preterm deliveries; this could be due to small (56 pregnant patients) study population.

We also analyzed the factors that affected perforation of the appendix and postoperative complications in acute appendicitis including pregnancy status of patient. However, we did not find that pregnancy status of patient had any influence. However we observed that, besides long patient time to surgery, WBC > 18000/mm^3^ was a risk factor associated with appendicular perforation and postoperative complications. This agrees with what has been reported in the literature [25, 26] even though some studies described no association between patient time to surgery and perforation [27].

As we are based in the developing world, we have to face several problems like scarcity of trained manpower and training opportunities, frequent usage of disposable surgical tools, shortage of funds to preserve equipment, and poor postoperative care. Besides that, due to poor economy, poor transportation services, and long distances in rural setting, the general public often have poor access to medical services. Mostly our patients and their families hesitate regarding the safety of surgery during pregnancy and do not realize the advantage due to lack of medical knowledge, poor education, and social stigma.

Regardless of all, we believe that this is the first descriptive comparative study of acute appendicitis in a pregnant and a nonpregnant cohort in the developing world. The limitations of our study were a retrospective, single centered design and small pregnant study population. The study and control groups were identified within two different time periods, 2011–2016 for study group and 2014–2016 for the control group which may have caused a bias. We acknowledge our limitation of not being able to compare certain preoperative data like BMI which was not mentioned in medical records of patients. Therefore, in order to verify our findings, additional appropriately designed prospective study in the developing world is suggested.

## 5. Conclusion

We did not find any differences in clinical presentation and postoperative outcomes of acute appendicitis in pregnant and nonpregnant patients. Hence similar perioperative treatment protocol can be followed for pregnant and nonpregnant groups even in resource-poor setting.

## Figures and Tables

**Table 1:** Preoperative data.

Variable	Pregnant (*n* = 56)	Nonpregnant (*n* = 164)	*P* ^*∗*^ value
Age (years)	26 (19–37)	26 (18–43)	0.35
Comorbidity, %	11 (19.6)	28 (17.1)	0.66
Cardiovascular disease	1 (6.4)	4 (2.4)	
Diabetes mellitus	2 (3.6)	5 (3.0)	
Respiratory diseases	1 (5.1)	3 (1.8)	
Anemia	5 (8.9)	10 (6.1)	
Hypertension	1 ( 1.8)	3 (1.8)	
Neurological problems	1 (1.8)	3 (1.8)	
Nausea/vomiting			0.72
Yes	38 (67.9)	107 (65.2)	
No	18 (32.1)	57 (34.8)	
Fever			0.65
Yes	23 (41.1)	73 (44.5)	
No	33 (58.9)	91 (55.5)	
Tenderness			0.86
Localised	45 (80.4)	130 (79.3)	
Generalised	11 (19.6)	34 (20.7)	
White blood cell count			0.93
Normal	21 (37.5)	58 (35.4)	
<18000/mm^3^	24 (42.9)	75 (45.7)	
>18000/mm^3^	11 (19.6)	31 (18.9)	
Ultrasonography			0.98
Positive	25 (44.6)	73 (44.5)	
Negative	31 (55.4)	91 (55.5)	

Categorical variables are presented as *n* (%); continuous variables are presented as median (interquartile range); ^*∗*^*P* value significant if < 0.05.

**Table 2:** Intraoperative and postoperative data.

Variable	Pregnant (*n* = 56)	Nonpregnant (*n* = 164)	*P* ^*∗*^ value
Time to surgery (hours)	36 (12–70)	30 (6–70)	0.28
Time to surgery (>48 hours)	18 (32.1)	51 (31.1)	0.88
Operative time (minutes)	50 (30–80)	50 (30–80)	0.19
Operative time (>60 minutes)	7 (12.5)	14 (8.5)	0.38
Pathology			0.52
Normal	12 (21.4)	35 (21.3)	
Inflamed	15 (26.8)	41 (25.0)	
Suppurative	26 (46.4)	78 (47.6)	
Gangrenous	3 (5.4)	10 (6.1)	
Perforation			
Yes	14 (25.0)	39 (23.8)	0.85
No	42 (75.0)	125 (76.2)	
Complications%	16 (28.6)	44 (26.8)	0.80
SSI^*♦*^	8 (14.3)	22 (13.4)	
LRTI^¶^	4 (7.1)	7 (4.3)	
UTI^†^	2 (3.6)	7 (4.3)	
Intraabdominal abscess	1 (1.8)	4 (2.4)	
Superficial thrombophlebitis	1 (1.8)	4 (2.4)	
Length of hospital stay (days)	5 (3–14)	4.5 (3–18)	0.36

Categorical variables are presented as *n* (%); continuous variables are presented as median (interquartile range); ^*♦*^SSI: surgical site infection; ^¶^LRTI: lower respiratory tract infection; ^†^UTI: urinary tract infection; ^*∗*^*P* value significant if <0.05.
